# Exercise Questions on Chapter Seventh

**Published:** 1884-01

**Authors:** 


### EXERCISE QUESTIONS ON CHAPTER SEVENTH.

---

What is the atomic weight of bromine?
What is its quantivalence?
When does bromine occur?
Is it found free?
Who discovered it?—when and where?
Are there any minerals that contain bromine?
Why did it receive the name of bromine?
Describe the whole method of its preparation?

---

SALICYLATE of zinc is highly spoken of as an astringent and antiseptic. An injection of a one half to a one per cent. solution has been successful in gonorrhœa. It is said to be superior to sulphate of zinc in ophthalmic affections.

